# Exercise Questions on Chapter Sixth

**Published:** 1883-12

**Authors:** 


### EXERCISE QUESTIONS ON CHAPTER SIXTH.

In how many classes, and what, are the non-metals arranged?

Name the elements of class first?

How many groups in first class?

Why as many groups as elements in this class?

Are hydrogen and oxygen placed in class first because of their chemical similarities?

Why are they so arranged?

Name the elements of the second class by their groups?

Which is the most important of the five groups given?

What is the basal principle of this classification?

What is $K_2 HAsO_3$?

Can there be such a compound as $P(NO_3)_3$?

Why?

What group name has been given to first group of class second?

What does halogen mean, and how derived?

Name the halogen group in the order of their chemical activity?

What is the quantivalence of this group?

Name some of the most striking characteristics of this group?

Why does the name of each terminate in *ene?*

Why are some of the constants of fluorine marked hypothetical?

In what natural compound is fluorine found?

Describe fluor spar?

What important compound of fluorine with hydrogen?

How is HF prepared?

Give the equation of reaction?

For what is HF used?

Describe the process for etching on glass?

Is this gas poisonous?

What the antidote?

